# ASO Author Reflections: Remote Home Monitoring After Surgery: Focus on Feasibility for Older Cancer Patients

**DOI:** 10.1245/s10434-020-08751-9

**Published:** 2020-06-25

**Authors:** L. T Jonker, M. M. H. Lahr

**Affiliations:** 1 Department of Surgical Oncology, University Medical Center Groningen, Groningen, The Netherlands; 2 Department of Epidemiology, University Medical Center Groningen, Groningen, The Netherlands

## Past

In the past decade, remote home monitoring has emerged to monitor surgical patients in the vulnerable period after hospital discharge.^1^ In addition, the potential value of e-health for patients after oncological surgery has been recognized.^2^ Although older cancer patients represent the majority of the oncological population who are at high risk for developing postoperative adverse events,^3^ there is limited knowledge about monitoring following onco-geriatric surgery;^4^ however, the use of technologies required to support this form of monitoring might be a challenge for this older population.

## Present

In this observational feasibility study,^5^ we described the implementation process of our study and the feasibility of a remote home monitoring system for surgical cancer patients over 65 years of age. Thirty-seven patients used a mobile application and were connected to several smart devices to monitor physical activity and/or vital signs preoperatively until 3 months postoperatively. The results indicate that elderly oncological patients in the study setting considered postoperative home monitoring acceptable and usable. More than half of the approached patients wanted to participate, and most were compliant with the use of the system up to 3 months after surgery. It has to be considered that our information technology (IT) system was still under development during study implementation and data were not monitored in real-time, therefore no interventions succeeded abnormal monitoring findings.

## Future

The results of this study provide a valuable contribution to the discussion on the feasibility of monitoring systems for older and vulnerable populations. Future home monitoring systems should measure a various range of parameters, yet remain usable and acceptable for older and vulnerable patients. This is necessary to ensure high compliance and completion rates of the population with the perceived greatest benefit of monitoring. Finally, the integration of monitoring systems into existing health care systems should be well explored to secure feasibility for health care professionals.

## References

[1] Cleeland CS, Wang XS, Shi Q (2011). Automated symptom alerts reduce postoperative symptom severity after cancer surgery: a randomized controlled clinical trial. J Clin Oncol..

[2] Penedo FJ, Oswald LB, Kronenfeld JP, Garcia SF, Cella D, Yanez B (2020). The increasing value of eHealth in the delivery of patient-centred cancer care. The Lancet Oncol.

[3] Ommundsen N, Nesbakken A, Wyller TB (2018). Post-discharge complications in frail older patients after surgery for colorectal cancer. Eur J Surg Oncol.

[4] van der Meij E, Anema JR, Otten RH, Huirne JA, Schaafsma FG (2016). The effect of perioperative E-health interventions on the postoperative course: A systematic review of randomised and non-randomised controlled trials. PLoS One.

[5] Jonker LT, Plas M, de Bock GH, Buskens E, van Leeuwen BL, Lahr MMH (2020). Remote home monitoring of older surgical cancer patients: Perspective on study implementation and feasibility. Ann Surg Oncol.

